# New Feature: Student and Trainee-Focused Podcast Interviews with Article Authors

**DOI:** 10.1016/j.advnut.2024.100234

**Published:** 2024-05-17

**Authors:** Steven A Abrams

**Affiliations:** Department of Pediatrics, Dell Medical School at the University of Texas at Austin, Austin, TX, United States

I am pleased to announce that *Advances in Nutrition: An International Review Journal* is introducing a new podcast-based feature to our Journal. We will be publishing freely available, ∼10-min audio podcasts based on recently published articles in our Journal. The podcasts will feature interviews between graduate students and faculty authors of articles. We anticipate initially publishing ∼1 a month, with a goal of increasing to 2 a month after a year or so as we learn more about the best way to present and publish these.

A key feature of our approach is that the intent is not a “review of the review” article but rather a discussion between the graduate student and the faculty about the topic in the review article and where the faculty perceives the field to be moving in terms of future research and innovation. Our goal is to stimulate thought and excitement among students and trainees at all levels, from college students through postdoctoral fellows, about the research topics being reviewed and hopefully to encourage them to read the original article and perhaps further pursue this field in their own research.

We would like to create podcasts that can be used in the classroom or reviewed by trainees as they go about traveling or exercising. The short format is intended to make this feasible for a range of uses. Podcast links can be found on the journal website including an introductory podcast about the series [1].

In the initial article-based podcast, graduate student Anahi Ramos-Gonzalez in the Department of Nutritional Sciences at the University of Texas at Austin discusses with Dr. Juan Rivera Dommarco, senior faculty at Instituto Nacional de Salud Pública in Mexico the approaches that have shown success in combating obesity, especially among children in Mexico [2]. What is especially exciting about this podcast is that there are separate but essentially identical versions of this podcast in English and Spanish, a new feature for ASN journals to have Spanish-language podcasts.

The second podcast is a discussion about the critical role of properly assessing growth in premature infants conducted by graduate student Madilyn Bradley in the Department of Nutritional Sciences at the University of Texas at Austin with Dr. Tanis Fenton, the esteemed dietitian and epidemiologist in Alberta, Canada, and lead in developing and updating the “Fenton Preterm Growth Charts” [3] for neonatal growth used in Neonatal Intensive Care Unit settings throughout the world. Consideration of issues related to assessing preterm growth were recently published by Dr. Fenton’s group in our Journal [4].

Our third podcast, to be available soon, will be a discussion between a graduate student and Dr. Jaclyn Albin, a physician and educator at the University of Texas at Southwestern, discussing the challenges of training medical students and physicians in evidence-based nutritional practice as reviewed in a recent article by Albin et al. [5]. Dr. Albin is the Director of the UT Southwestern Culinary Medicine Program [6] and specializes in both internal medicine and pediatrics. Her efforts have focused on the critical role of education at all levels in sound nutrition practices.

As we initiate this new feature, we will not be accepting externally submitted interviews at this time but may consider this in the future. Authors of manuscripts are welcome to email the journal if they would like to have us consider doing a podcast with them about their article, which would be conducted around the time of the article’s final acceptance.

We hope that you enjoy this new feature and welcome feedback on this approach and further development of podcasts within our Journal.

## Author contribution

The sole author was responsible for all aspects of this manuscript.

## Conflicts of interest

The author reports no conflicts of interest.

## Funding

The author reported no funding received for this work.

## References

[1] Podcast landing page https://advances.nutrition.org/multimedia/audio.

[2] Rivera J.A., Colchero M.A., Pérez-Ferrer C., Barquera S. (2024). Perspective: Mexico's experience in building a toolkit for obesity and noncommunicable diseases prevention. Adv. Nutr..

[3] University of Calgary, Welcome to the Fenton preterm growth chart site. [Internet]. [cited April 10, 2024]. Available from: https://live-ucalgary.ucalgary.ca/resource/preterm-growth-chart/preterm-growth-chart.

[4] Fenton T.R., Barr S.M., Elmrayed S., Alshaikh B. (2024). Expected and desirable preterm and small infant growth patterns. Adv. Nutr..

[5] Albin J.L., Thomas O.W., Marvasti F.F., Reilly J.M. (2024). There and back again: a 40-year perspective on physician nutrition education. Adv. Nutr..

[6] University of Texas Southwestern, The culinary medicine program at UT Southwestern. [Internet]. [cited April 10, 2024]. Available from: https://culinarymedicine.org/culinary-medicine-partner-schools/partner-medical-schools/culinary-medicine-southwestern/.

